# A case of sudden onset of thyroid storm just before cesarean section manifesting congestive heart failure and pulmonary edema

**DOI:** 10.1186/s40981-017-0088-3

**Published:** 2017-04-26

**Authors:** Yuki Sugiyama, Ryusuke Tanaka, Yuki Yoshiyama, Takashi Ichino, Norimasa Hishinuma, Sari Shimizu, Noriko Imai, Kunihiro Mitsuzawa, Mikito Kawamata

**Affiliations:** Department of Anesthesiology and Resuscitology, Shinshu University School of Medicine, 3-1-1, Asahi, Matsumoto, Nagano 390-8621 Japan

**Keywords:** Thyroid storm, Pulmonary edema, Ritodrine, MgSO_4_, Mitral regurgitation

## Abstract

**Background:**

Since acute respiratory failure (ARF) is a life-threatening complication, particularly in the gestational period, differential diagnosis and rapid treatment are required. Among the various causes of sudden onset of ARF, thyroid storm is a rare cause in a parturient complicated with well-controlled hyperthyroidism. In this case report, we describe a parturient with hyperthyroidism in whom a thyroid storm manifesting congestive heart failure and pulmonary edema developed just before an emergency ceasarean section, even though hyperthyroidism was well-controlled with antithyroid drugs.

**Case presentation:**

A 36-year-old pregnant woman was diagnosed as having clinical chorioamnionitis, and an emergency cesarean section was performed at 25 weeks of pregnancy. She had a complication of hyperthyroidism accompanied by mild mitral regurgitation, and she had been treated with methimazole. She was treated with ritodrine and MgSO_4_ for the threat of premature delivery. At the preoperative consultation, her percutaneous oxygen saturation (SpO_2_) was 98% on room air. When she was admitted to the operating room, her heart rate and blood pressure were 130 beats/min and 196/78 mmHg, respectively. SpO_2_ was 88% on room air without any symptoms; however, just after starting oxygen administration via a facemask, she complained of severe respiratory distress and became agitated. Partial pressure of arterial oxygen was 108 mmHg with an inspiratory oxygen fraction of 1.0. Chest radiography revealed pulmonary congestion, and transesophageal echocardiography revealed normal right ventricular function without an embolus and severe mitral regurgitation with preserved left ventricular function. Contrast-enhanced computed tomography after the operation revealed no pulmonary embolus but revealed a pulmonary effusion, and free triiodothyronine level was increased at the onset of dyspnea. Therefore, we diagnosed the causes of sudden onset of dyspnea as pulmonary edema and congestive heart failure induced by a thyroid storm.

**Conclusion:**

Sudden onset of a thyroid storm just before a cesarean section occurred in a patient with several risk factors of thyroid storm and pulmonary edema, including pregnancy, treatment with tocolytic agents, and infection. The involvement of these multiple factors was considered to be the cause of the sudden onset of the thyroid storm and the cause of rapidly progressive pulmonary edema.

## Background

Acute respiratory failure (ARF) during the gestational period is sometimes life-threatening, and early differential diagnosis and rapid treatment are required. Pulmonary edema, pulmonary embolism, and peripartum cardiomyopathy are critically important causes of ARF, and the onset pattern may be useful in making the differential diagnosis. Among these diseases, pulmonary edema is usually considered to have a gradual onset; however, we experienced rapidly progressive pulmonary edema in a parturient complicated with well-controlled hyperthyroidism just before an emergency ceasarean section. The cause of the rapidly progressive pulmonary edema was considered to be sudden onset of a thyroid storm, which is rare, especially in patients with well-controlled hyperthyroidism.

## Case presentation

**Fig. 1:**
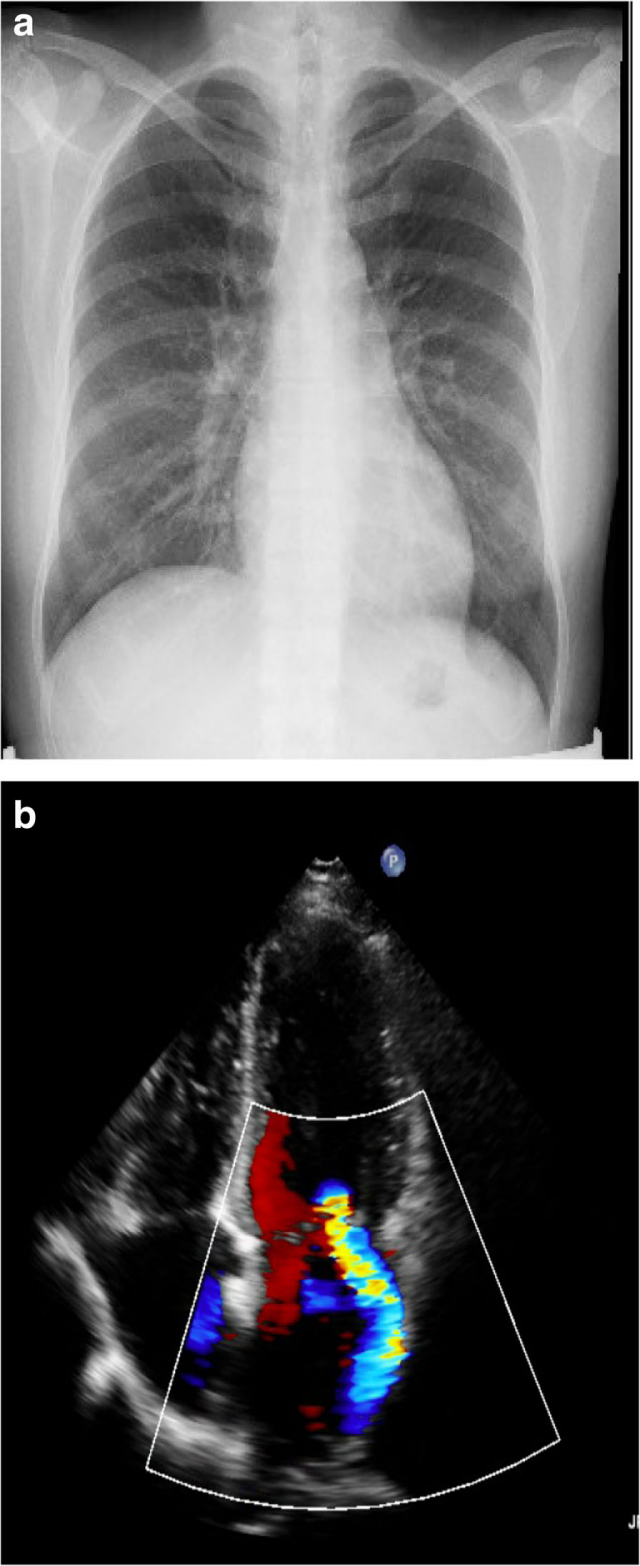
Preoperative examinations. **a** Chest radiograph showing no abnormality. **b** Four-chamber view of a transthoracic echocardiogram showing mild mitral regurgitation with tethering

A 36-year-old pregnant woman was diagnosed as having clinical chorioamnionitis, and an emergency cesarean section was performed at 25 weeks of her second pregnancy. At 16 weeks of gestation, she was diagnosed as having hyperthyroidism for the first time. She presented with proptosis and occasional sweating and palpitations. Her heart rate (HR) and blood pressure (BP) were 100 beats/min and 112/65 mmHg, respectively. Her goiter was visible and palpable. The levels of thyroid-stimulating hormone, free triiodothyronine (T3), and free thyroxine (T4) were 0.005 μU/ml (normal range, 0.34–3.5 μU/ml), 16.25 pg/ml (2.0–4.0 pg/ml) and 2.62 ng/dl (1.0–2.0 ng/dl), respectively. Although chest radiography revealed no abnormality (Fig. 1a), transthoracic echocardiography revealed mild mitral regurgitation (MR) with tethering (Fig. 1b). Treatment with propylthiouracil (900 mg/day orally) was started. At 22 weeks of gestation, she was admitted to undergo treatment for threatened premature delivery, which started with ritodrine (50 μg/min) and MgSO_4_ (1.1 g/h) from 23 weeks of gestation. The treatment for hyperthyroidism was switched from propylthiouracil (900 mg/day orally) to methimazole (90 mg/day orally), following the recommendation of guidelines [1, 2]. At 24 weeks of gestation, the doses of the tocolytic agents were changed to 42 μg/min for ritodrine and 1.6 g/h for MgSO_4_. Her thyroid hormone level was evaluated every week. One day before the surgery, free T3 level was 5.63 pg/mL and free T4 level was 2.38 ng/dL. The methimazole dose was then reduced to 60 mg/day.

At 25 weeks and 0 days of gestation, an emergency cesarean section was performed since she developed fever with body temperature of up to 39 °C and clinical signs of infection and was diagnosed as having clinical chorioamnionitis. At the preoperative consultation on the day of surgery, HR was 100 beats/min and BP was 122/78 mmHg. She did not complain of respiratory distress, and percutaneous oxygen saturation (SpO_2_) was 98% in room air. The fetus was in good condition. Therefore, we planned a cesarean section under combined spinal-epidural anesthesia (CSEA).

**Fig. 2:**
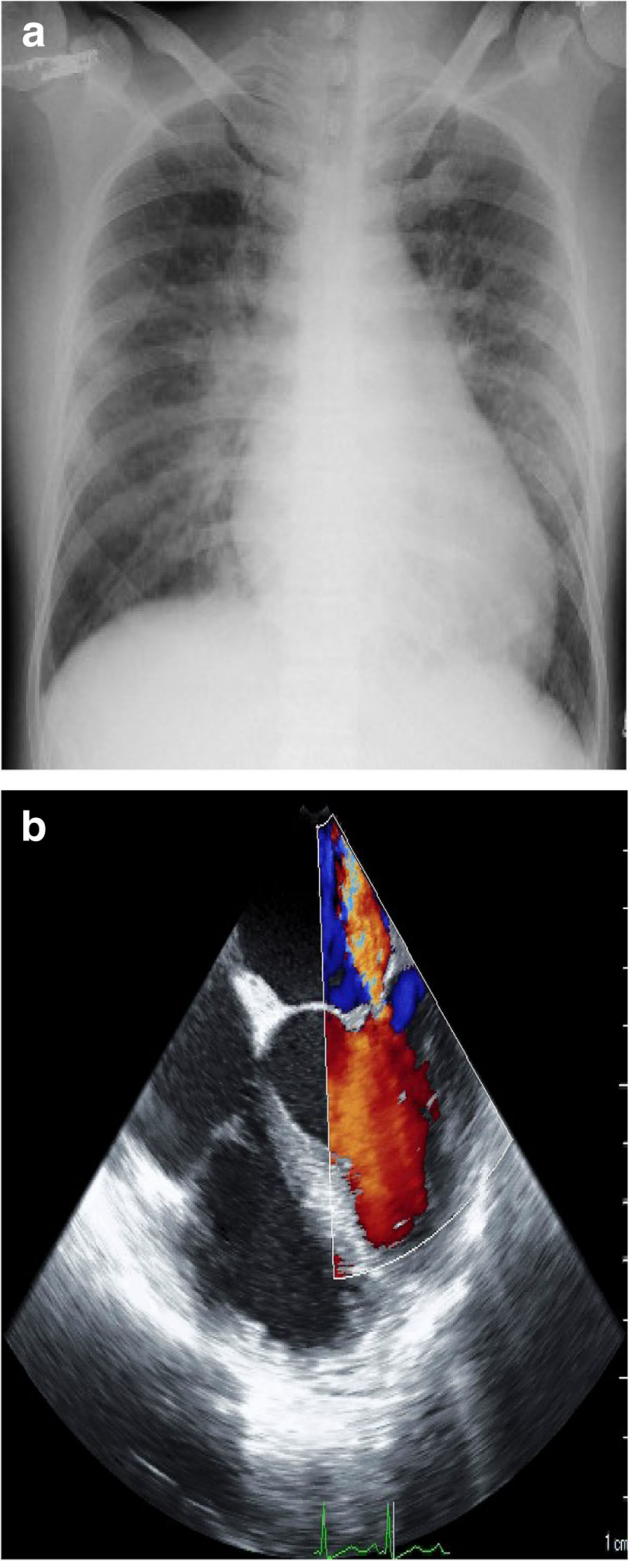
Examinations after exacerbation of dyspnea. **a** Chest radiograph showing cardiac enlargement and bilateral pulmonary infiltration. **b** Four-chamber view of a transesophageal echocardiogram showing severe mitral regurgitation

During transfer from the ward to the operating room, her vital signs were not monitored. Thirty minutes after preoperative consultation, she could move from the stretcher to the operating table by herself without any problem and without any complaint of dyspnea, and then we started monitoring her vital signs. HR, BP, and SpO_2_ were 130 beats/min, 196/78 mmHg, and 88% on room air, respectively. Thus, oxygen was immediately administered at 10 L/min via a facemask. Just after that, she complained of severe respiratory distress and became agitated. SpO_2_ was 92% with 10 L/min oxygen administration. An arterial catheter was inserted, and arterial blood gas analysis revealed partial pressure of arterial oxygen (PaO_2_) of 108 mmHg, partial pressure of carbon dioxide of 24.2 mmHg, and pH of 7.513 with a fraction of inspiratory oxygen (FiO_2_) of 1.0. Chest radiography revealed bilateral pulmonary congestion (Fig. 2a). Examination by transthoracic echocardiography was impossible because the patient was struggling against dyspnea and she was unable to maintain a stable position. Mask-assisted ventilation was inadequate for the same reason, although oxygenation continued to exacerbate. To improve her respiratory status, we decided to sedate her as soon as possible, and we therefore changed the anesthesia plan from CSEA to general anesthesia. Twenty minutes after entering the operating room, rapid sequence induction was attempted with 120 mg of propofol and 80 mg of rocuronium. Simultaneously with the inhibition of spontaneous breathing, SpO_2_ rapidly decreased to 75%, requiring assisted mask ventilation. One minute later, her trachea was intubated, and the cesarean section was started. Her SpO_2_ increased to 100% with FiO_2_ of 1.0, and a male baby was delivered 3 min after the start of surgery. His Apgar score was 5 at 1 min and 7 at 5 min. She was ventilated via volume-controlled ventilation, at a tidal volume of 6–8 ml/kg, peak inspiratory pressure of less than 30 cmH_2_O and positive end-expiratory pressure of 5–7 cmH_2_O in accordance with a lung protective strategy.

After parturition, transesophageal echocardiography was immediately performed by a cardiologist and revealed normal right ventricular function without an embolus and severe MR with preserved left ventricular function (ejection fraction >60%) (Fig. 2b). Within 30 min after the start of surgery, HR and BP gradually decreased from 130 to 110 beats/min and from 196/78 to 110/55 mmHg, respectively. Consistent with the decrease in BP, the MR gradually improved and the PaO_2_/FiO_2_ ratio increased to 187.

After the operation, contrast-enhanced computed tomography (CT) was performed and revealed no pulmonary embolus, but pulmonary effusion was present. Laboratory data obtained immediately after the dyspnea showed the following values with their normal ranges: D-dimer, 5.3 μg/mL (<1 μg/ml); N-terminal B-type natriuretic peptide (NT-pro BNP) 616.7 pg/mL (<125 pg/mL); zinc coproporphyrin-1, < 1 pmol/mL (<1.6 pmol/mL); and sialyl-Tn antigen, 20 U/mL (<46 IU/mL). Free T3 level was 8.98 pg/mL and free T4 level was 2.28 ng/dL. She was transferred to the intensive care unit and treated with intermittent administration of furosemide at 10 mg. Her oxygenation gradually improved and her trachea was extubated on postoperative day 1. At the postoperative interview, she said that she had a panic-like feeling in the operating room. She was discharged from the intensive care unit on postoperative day 1. On postoperative day 7, transthoracic echocardiography revealed that the MR was trivial. Her clinical course was uneventful thereafter, and she was discharged on postoperative day 11, continuing methimazole therapy at 20 mg/day.

### Discussion

This case shows the importance of a thyroid storm as a differential diagnosis of ARF, even if respiratory symptoms rapidly progresses. In pregnancy, ARF can be caused by various diseases, and both echocardiography and contrast-enhanced CT are indispensable for a definite diagnosis. We could not examine her via transthoracic echocardiography before general anesthesia because the patient was struggling against dyspnea and she was unable to maintain a stable position; however, if possible, it is better to make the diagnosis before general anesthesia. In our situation, we considered that general anesthesia could improve her respiratory condition and a rapid diagnosis could be reached via transesophageal echocardiography.

We initially assumed that the cause of ARF was pulmonary embolism because of its sudden onset; however, intraoperative transesophageal echocardiography revealed no right ventricular load and preserved left ventricular function. In addition, postoperative contrast-enhanced CT and laboratory examination revealed no evidence of pulmonary embolism. From these findings, the cause of ARF in this case was considered to be pulmonary edema induced by congestive heart failure resulting from exacerbated MR caused by tachycardia and increase of afterload.

The prevalence of pulmonary edema in pregnant women has been reported to be 0.08%, and preeclampsia, congestive heart failure, hyperthyroidism, administration of tocolytic agents, and fluid overload are known to be major causes of pulmonary edema [3, 4]. Hyperthyroidism occurs in 0.2% of pregnant women [3] and is frequently accompanied by MR. A report showed that moderate MR was present in 13% of patients with hyperthyroidism [5]. Papillary muscle dysfunction has been proposed as the possible mechanism of MR complicated with hyperthyroidism, and MR is curable after treatment of the hyperthyroidism [5]. At diagnosis, our patient’s thyroid hormone levels were high (16.25 pg/mL for free T3 and 2.62 ng/dL for free T4), and mild MR appeared as a complication. Thus, treatment was started, and her thyroid hormone levels were lowered to the recommended levels [1, 2] on the day before surgery. However, free T3 level was increased at the onset of dyspnea, and other symptoms such as agitation, tachycardia, and congestive heart failure were present. We therefore considered that rapidly progressive pulmonary edema was caused by sudden onset of a thyroid storm.

A thyroid storm is often triggered by severe physical or mental stress. Criteria for the diagnosis of thyroid storm are (1) presence of thyrotoxicosis (elevated free T3 and/or thyroxine T4 levels) and at least one central nervous system (CNS) manifestation plus one of the following: fever (38 °C or higher), tachycardia (130 beats/min or faster), congestive heart failure, or gastrointestinal/hepatic manifestations, or (2) presence of thyrotoxicosis and three or more of the manifestations just listed other than CNS manifestations [6]. In our case, it was difficult to determine whether the symptoms (fever, tachycardia, agitation, congestive heart failure) were caused by a thyroid storm or were simply symptoms of underlying diseases that are possibly triggered by a thyroid storm. In such a situation, the symptoms should be regarded as secondary to thyroid storm [6]; therefore, a thyroid storm occurred in this case and it was considered to be mainly triggered by infection. Drugs used for treatment of a thyroid storm to lower thyroid hormones are antithyroid drugs, inorganic iodine, and bile acid sequestrants, and other drugs to control the systemic manifestations are β-blockers, glucocorticoids, and acetaminophen [7]. Although we did not use these drugs for systemic manifestations because the vital signs and oxygenation improved without these drugs, it might have been better to use these drugs.

There are many case reports on thyroid storm [3, 8–14]. However, the clinical features described in those reports are different from the case reported here based on the following points. First, hyperthyroidism had been treated and controlled during pregnancy in our patient [3, 8, 9]. Second, acute pulmonary edema rapidly developed just before anesthetic induction; our patient had not received any anesthetic or undergone a surgical procedure [10, 14]. Third, our patient did not suffer from either cardiac failure or lethal arrhythmia. These clinical features of the thyroid storm that our patient experienced have not been described in previous reports [8, 9, 11–13]. Thus, we thought that other exacerbating factors might have played pivotal roles in the sudden manifestation of the symptoms of a thyroid storm and rapidly progressive pulmonary edema.

Ritodrine as a β-adrenergic agonist and MgSO_4_ were simultaneously used as tocolytic therapy at low doses; the recommended dose for ritodrine is 50–150 μg/min and that for MgSO_4_ is 1–2 g/h [15]. Ritodrine is a well-known cause of thyroid storm, and both ritodrine and MgSO_4_ are well-known causes of pulmonary edema induced by cardiovascular and/or renal changes that increase hydrostatic pressure [16]. The incidence rates of pulmonary edema caused by ritodrine and MgSO_4_ are thought to be approximately 0.25 and 8.5%, respectively [16, 17], and the risk of pulmonary edema significantly increases when a β-adrenergic agonist and MgSO_4_ are simultaneously administered.

Our patient developed fever due to chorioamnionitis, with a body temperature of up to 39 °C, on the day of surgery. Infection is also a known cause of thyroid storm and an exacerbating factor of pulmonary edema because it increases vascular permeability. Water content in the patient’s lungs was supposed to have risen at the time of the preoperative consultation, although she did not complain of dyspnea and SpO_2_ was 98% in room air. In this situation, emotional stress related to the emergency cesarean section and agitation induced by a thyroid storm occurred when entering the operating room and acted as a final trigger of rapidly progressive pulmonary edema. This mental stress might have induced the increase in the afterload and diastolic dysfunction by tachycardia followed by exacerbation of MR. The elevated levels of NT-pro-BNP might have reflected this cardiac overload, although hyperthyroidism itself also increases the level of NT-pro-BNP [18]. The multiple and overlapping risk factors of pulmonary edema and thyroid storm were considered to have predisposed the patient to rapidly progressive pulmonary edema.

## Conclusions

We treated a sudden onset of thyroid storm just before a cesarean section manifesting congestive heart failure and rapidly progressive pulmonary edema. This case indicates that in a patient with multiple risk factors, thyroid storm and pulmonary edema are likely to develop suddenly even if each preoperative complication was being carefully treated.

## Abbreviations

ARF: Acute respiratory failure
BP: Blood pressure
CNS: Central nervous system
CSEA: Combined spinal-epidural anesthesia
CT: Computed tomography
FiO_2_: Fraction of inspiratory oxygen
HR: Heart rate
MR: Mitral regurgitation
NT-pro-BNP: N-terminal B-type natriuretic peptide
PaO_2_: Partial pressure of arterial oxygen
SpO_2_: Percutaneous oxygen saturation
T3: Triiodothyronine
T4: Thyroxine
